# Precise location of linear epitopes on the capsid surface of feline calicivirus recognized by neutralizing and non-neutralizing monoclonal antibodies

**DOI:** 10.1186/s13567-020-00785-x

**Published:** 2020-05-01

**Authors:** Carolina Cubillos-Zapata, Iván Angulo, Horacio Almanza, Belén Borrego, María Zamora-Ceballos, José R. Castón, Ignacio Mena, Esther Blanco, Juan Bárcena

**Affiliations:** 1 Centro de Investigación en Sanidad Animal, INIA-CISA, Valdeolmos, Madrid, Spain; 2 Department of Structure of Macromolecules, Centro Nacional de Biotecnología/CSIC, Cantoblanco, Madrid, Spain; 3 Present Address: Innate Immunity Group, IdiPAZ Institute for Health Research, La Paz Hospital, 28046 Madrid, Spain; 4 Present Address: Facultad de Medicina y Psicología de la Universidad Autónoma de Baja California, Tijuana, Mexico; 5 Present Address: Department of Microbiology, Icahn School of Medicine at Mount Sinai, New York, USA

## Abstract

We report the generation, characterization and epitope mapping of a panel of 26 monoclonal antibodies (MAbs) against the VP1 capsid protein of feline calicivirus (FCV). Two close but distinct linear epitopes were identified at the capsid outermost surface (P2 subdomain) of VP1, within the E5′HVR antigenic hypervariable region: one spanning amino acids 431-435 (PAGDY), highly conserved and recognized by non-neutralizing MAbs; and a second epitope spanning amino acids 445-451 (ITTANQY), highly variable and recognized by neutralizing MAbs. These antibodies might be valuable for diagnostic applications, as well as for further research in different aspects of the biology of FCV.

## Introduction, methods and results

Feline calicivirus (FCV) has a worldwide distribution and is a major pathogen of domestic cats, belonging to the *Vesivirus* genus within the *Caliciviridae* (reviewed in [1, 2]). It causes a variety of clinical manifestations, such as upper respiratory tract disease, stomatitis and lameness. Highly virulent strains (VS-FCV), causing virulent systemic disease (VSD) leading to high mortality (40–60%), have been reported in North America and Europe [3].

**Figure 1:**
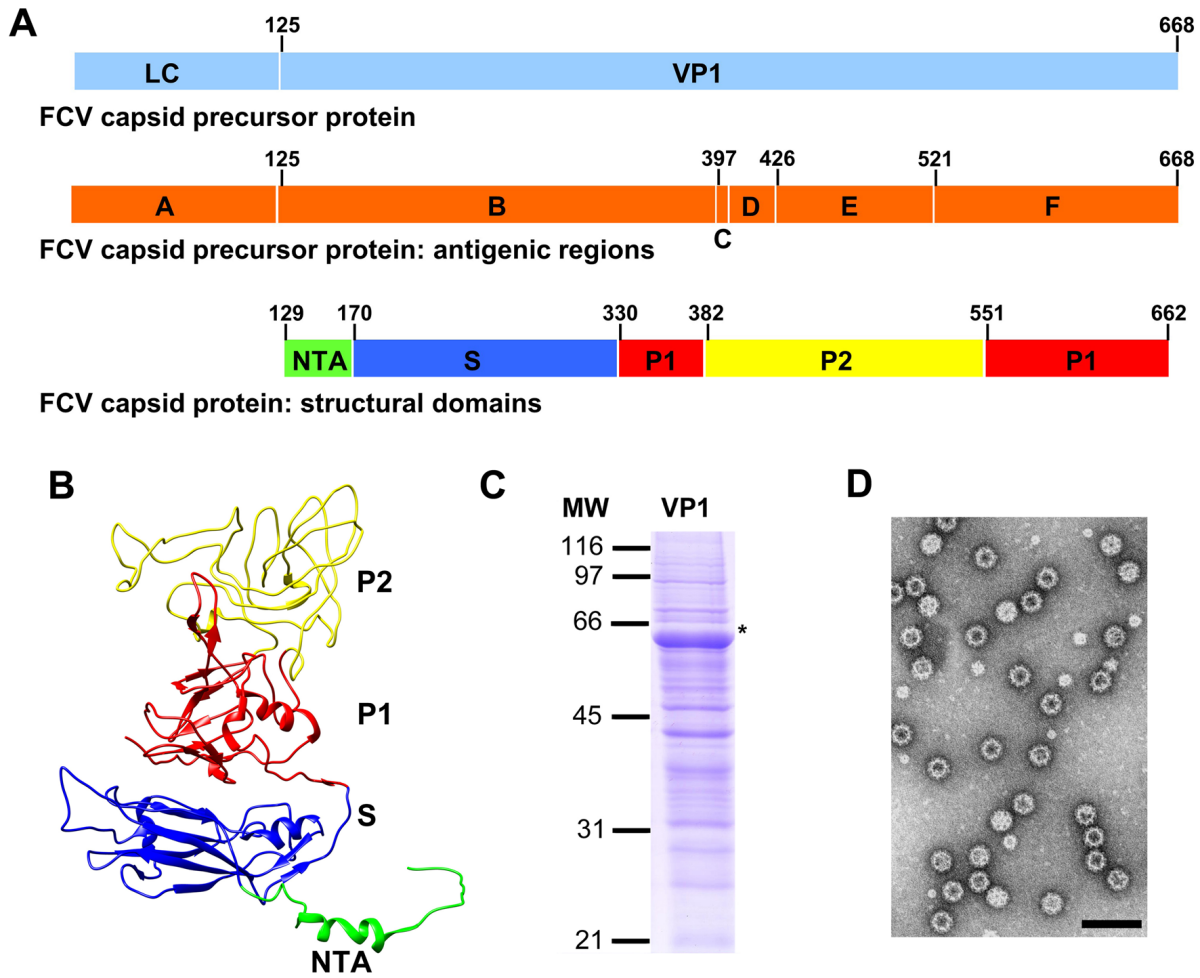
**Structure of FCV capsid protein, VP1. A** Schematic representation of FCV capsid precursor protein, which is cleaved into mature proteins LC and VP1. The figure shows capsid precursor protein antigenic regions (**A**–**F**) and the VP1 structural domains (NTA, S, P1 and P2). **B** Ribbon representation of the VP1 protein structure (Protein Data Bank [PDB] accession number 3M8L). The NTA, S domain, P1 and P2 subdomains are indicated. **C** Coomassie blue stained SDS-10% PAGE of H5 insect cell extracts infected with recombinant baculovirus expressing VP1 protein. Molecular weight markers (MW) are given on the left (× 103 Da). **D** Electron micrograph of a negatively stained sample of purified FCV VLPs. Bar, 100 nm.

The FCV genome is a positive-sense single stranded RNA (~7.6 kb) that contains three open reading frames (ORFs). ORF1 is located at the 5′ end of the genome and encodes the viral nonstructural proteins. ORF2 encodes the major capsid protein, VP1. ORF3 encodes a putative minor structural protein, VP2. A distinguishing feature unique to vesiviruses, in contrast to other caliciviruses, is the expression of the major capsid protein from ORF2 as a precursor protein (73–78 kDa), which is post-translationally cleaved into the leader capsid protein (LC) and the mature capsid protein of 60 kDa, VP1 (Figure 1A). On the basis of amino acid sequence alignment and antigenic analysis, the capsid precursor protein has been divided into six distinct regions, denoted as regions A–F, [4] (Figure 1A). Region A corresponds to the LC protein. Regions B, D, and F are relatively conserved among FCV isolates, whereas regions C and E are highly variable. Region E is considered immunodominant and has been further divided into 5′ and 3′ hypervariable regions (E5′HVR and E3′HVR), separated by a conserved central region (Econsv) [4, 5].

Caliciviruses are nonenveloped, icosahedral viruses sharing a common architectural framework. The capsid (~40 nm diameter) comprises 180 copies, organized as 90 dimers, of the single capsid subunit, VP1, arranged on a T = 3 icosahedral lattice [6–8]. The VP1 monomer has three structural domains (Figure 1B): An internally located N-terminal arm (NTA), a shell domain (S) forming a continuous scaffold, and a flexible protruding domain (P) at the capsid surface, which contains determinants for virus-host receptor interactions and antigenic diversity [5, 9, 10]. The P domain can be further divided into P1 and P2 subdomains, with P2 subdomain located at the outermost surface-exposed region of the viral capsid.

FCV is one of the few caliciviruses for which a protein receptor has been identified. Attachment and entry of FCV is mediated by feline junctional adhesion molecule A (fJAM-A), which binds to the outer face of the P2 subdomain of VP1 [8, 11–13].

Monoclonal antibodies (MAbs) are useful tools for analyzing antigenic properties of viruses. Panels of MAbs have been generated against FCV capsid protein, including neutralizing and non-neutralizing antibodies [14–17]. So far, epitopes recognized by MAbs to the FCV capsid have not been identified, although previous studies mapped the binding sites of linear neutralizing MAbs between amino acids 381 to 458 [14, 18] involving E5′HVR region. In addition, sequence analysis of MAb neutralization-resistant variants clustered point mutations disrupting linear neutralizing epitopes to the E5′HVR region and conformational neutralizing epitopes to the E3′HVR region [15], both within P2 subdomain.

Here we report the generation and characterization of a panel of MAbs against VP1. Most of the MAbs recognized antigenic region E. Two close linear epitopes were identified located within the 35 amino acid long E5′HVR region, one recognized by non-neutralizing and the other recognized by neutralizing MAbs.

We used virus like particles (VLPs) as immunogen for the generation of FCV-specific MAbs, following an approach we had successfully used before to raise MAbs directed against the capsid protein of other caliciviruses, such as swine norovirus [19] and rabbit hemorrhagic disease virus (RHDV) [9]. Briefly, we generated a recombinant baculovirus (BacPAK baculovirus expression system, Clontech) harbouring the coding sequences of mature proteins VP1 and VP2, and the 3′ untranslated region of FCV (Urbana strain GenBank accession no. L40021), following procedures described before [20, 21]. Cultures of H5 insect cells were infected with the recombinant baculovirus to analyze the expression of the recombinant FCV capsid protein. A major polypeptide band with the expected molecular mass of ~60 kDa was identified after analysis by SDS-10% PAGE (Figure 1C, asterisk). Electron microscopy analysis of negatively stained preparations of purified recombinant FCV capsid protein revealed virus-like particles (VLPs) with similar size and morphology to authentic FCV virions (Figure 1D). These FCV VLPs were subsequently used to immunize BALB/c mice in order to obtain FCV VP1-specific MAbs, following previously described procedures [19]. After screening of the hybridoma cell lines, 26 positive clones derived from four different immunized mice were selected.

**Figure 2:**
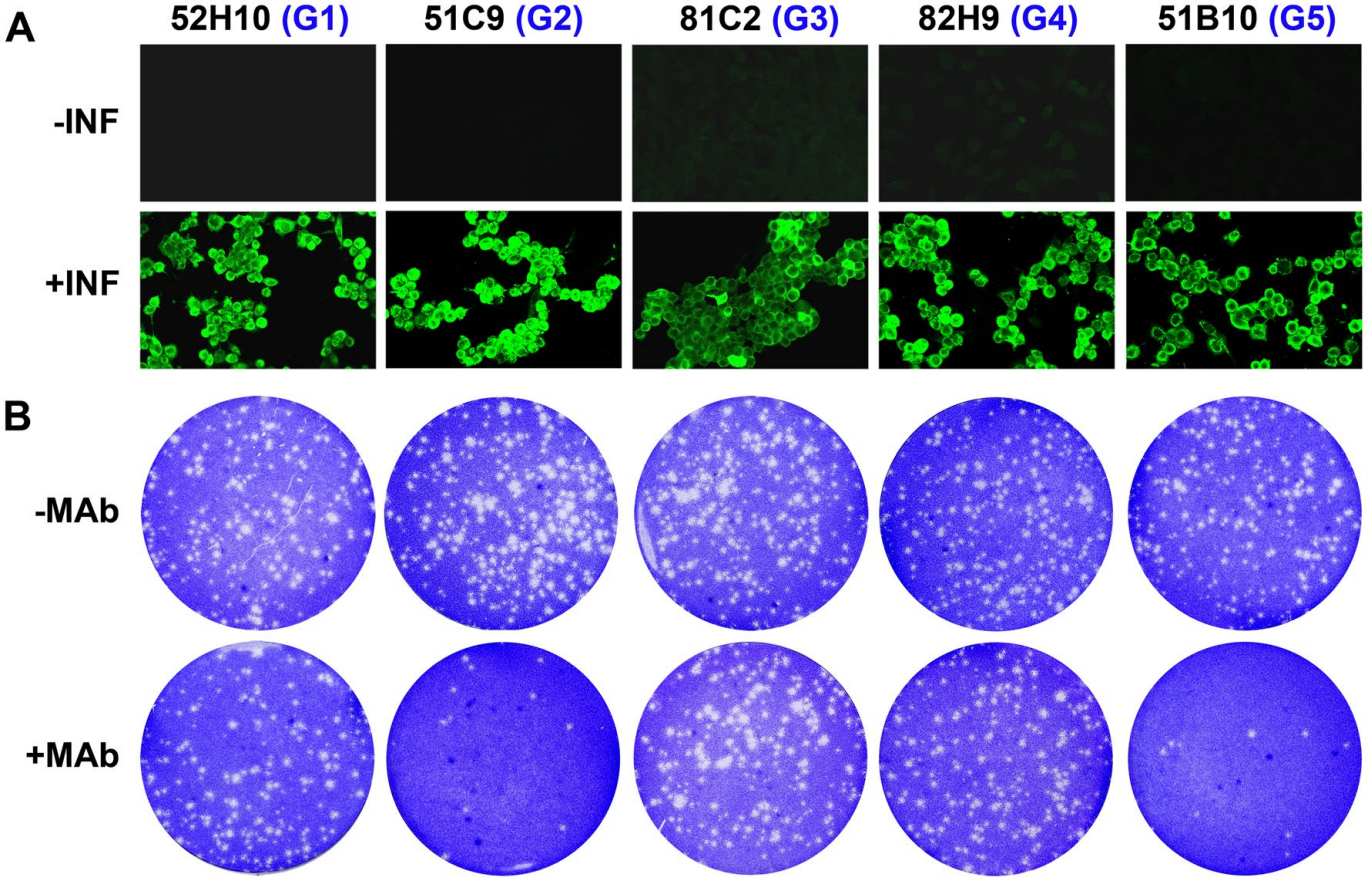
**Characterization of MAbs against FCV VP1 protein.** The figure shows results obtained with MAbs representative of each of the five different antibody groups, G1-G5. **A** Indirect immunofluorescence assays performed using non-infected (−INF) or FCV-infected CRFK cells (+INF). **B** Plaque-reduction neutralization assay specific for FCV virus, performed as previously described [22]. The FCV virus was preincubated in the absence (-MAb) or presence (+MAb) of MAbs (hybridoma supernatants diluted 1/20).

The MAbs were characterized by analyzing the reactivity of the corresponding hybridoma supernatants in ELISA, Western blot and immunofluorescence assays. All the MAbs reacted positively with purified FCV VLPs in indirect ELISA assays (data not shown). The 26 MAbs also reacted positively in immunofluorescence assays with FCV-infected feline CRFK cells (Figure 2A), demonstrating that the MAbs raised against recombinant VP1 protein (VLPs), recognized the FCV capsid protein expressed in the context of a FCV virus infection. 22 of the 26 MAbs reacted positively in Western blot assays both, against purified FCV VLPs and VP1 protein present in FCV-infected CRFK cell-extracts ([22] and data not shown), suggesting these MAbs recognized linear epitopes. Accordingly, the same 22 MAbs recognized the FCV capsid protein in indirect ELISA assays in which the antigen had been previously denatured by treatment with 7 M urea, before adsorption to the plates. In contrast, the remaining four MAbs did not react in Western blot or in indirect ELISA assays using urea-treated FCV capsid protein, indicating that these MAbs recognized conformational epitopes.

Subsequently, the panel of MAbs was analyzed for neutralizing activity using a plaque-reduction assay specific for FCV virus, as previously described [22]. 12 MAbs exhibited significant levels of anti-FCV neutralizing activity (>90% plaque reduction) while the rest of the MAbs was shown to be non-neutralizing (Figure 2B; in row +MAb, compare 2^nd^ and 5^th^ plates with 1^st^, 3^rd^ and 4^th^ plates).

**Figure 3:**
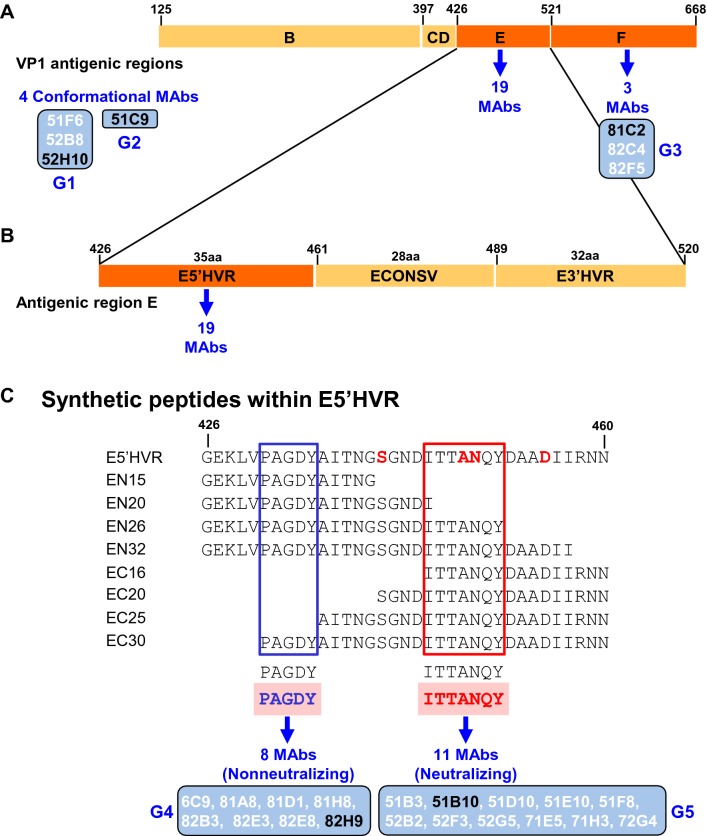
**Fine mapping of the epitopes recognized by the MAbs. A** Schematic representation of VP1 antigenic regions (**B**–**F**). **B** Antigenic region E, which is further divided into E5′HVR, Econsv and E3′HVR regions. **C** Set of overlapping synthetic peptides encompassing truncated sequences within E5′HVR region. Based on the epitope mapping and neutralizing activity analyses performed, the 26 MAbs reported in this study were classified into five groups, G1-G5 (blue boxes). The MAbs representative of each group, shown in Figure 2, are depicted in black in this figure. Amino acids highlighted in red in E5′HVR sequence indicate previously described MAb escape mutations at positions 441, 448, 449 and 455 [15].

As a next step in the characterization of the MAbs, we performed an epitope mapping analysis, which was carried out in subsequent steps, by analyzing MAb reactivities with sets of truncated FCV capsid polypeptides and synthetic peptides (Figure 3). First, truncated polypeptides corresponding to the FCV capsid protein antigenic regions: B, CD, E and F were expressed in *E. coli* as recombinant glutathione S-transferase (GST) fusion proteins (Figure 3A). Next, the amino acid sequences of antigenic region E: E5′HVR, Econsv and E3′HVR were incorporated as foreign antigens and displayed by chimeric RHDV VLPs, using procedures previously described [22, 23] (Figure 3B). Finally, a set of overlapping synthetic peptides encompassing truncated sequences within E5′HVR region was designed (Figure 3C). The different sets of truncated capsid polypeptides and synthetic peptides prepared were used as antigens in indirect ELISA assays to analyze their reactivity with the panel of FCV MAbs. The results obtained are summarized in Figure 3. Based on the epitope mapping and neutralizing activity analyses performed, the MAbs could be classified into five different groups, G1-G5 (Figures 2 and 3). Group G1, represented by MAb 52H10 in Figure 2, included three non-neutralizing conformational MAbs. Group G2 consisted of conformational MAb 51C9, exhibiting neutralizing activity. The conformational MAbs did not react with any of the truncated polypeptides. Thus, the location of their epitopes could not be determined. Group G3, represented by 81C2 in Figure 2, included three non-neutralizing MAbs recognizing epitopes within region F (Figure 3A). The epitopes recognized by the remaining 19 MAbs, including non-neutralizing ones like 82H9 and neutralizing MAbs like 51B10 (Figure 2), were all mapped to the 35 amino acid region E5′HVR (aa 426 to 460) (Figure 3B). Subsequently, screening of the overlapping peptide set enabled the identification of two close but distinct linear epitopes: an epitope spanning amino acids 431–435 (PAGDY), recognized by 8 non-neutralizing MAbs (group G4), and an epitope spanning amino acids 445-451 (ITTANQY), recognized by 11 neutralizing MAbs (Figure 3C).

**Figure 4:**
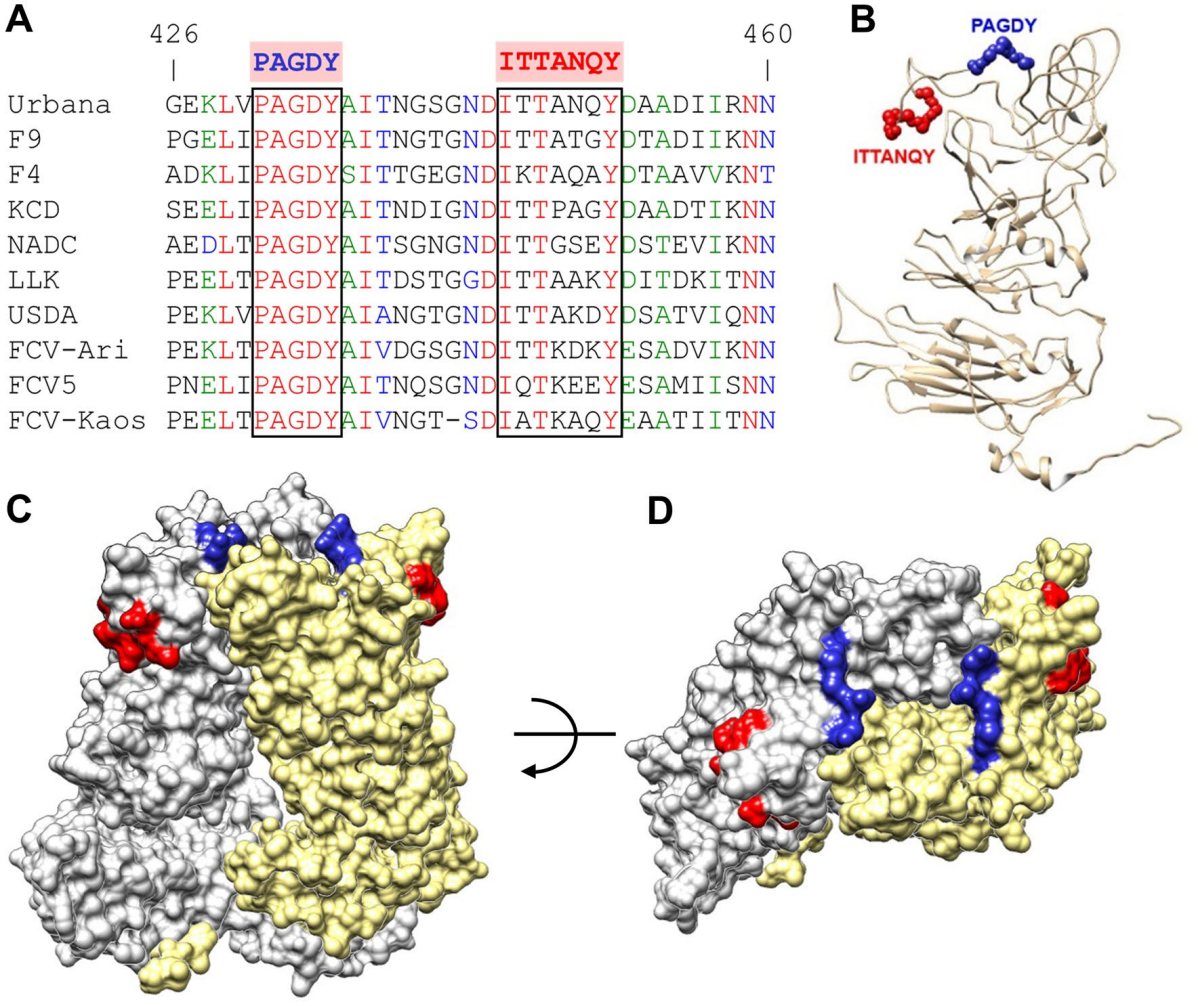
**Sequence analysis and epitope location. A** Amino acid sequence alignment of the E5′HVR region of 10 representative FCV strains. The sequences corresponding to the epitopes spanning aa 431-435 (PAGDY) and aa 445-451 (ITTANQY) in Urbana strain are indicated. **B** to **D** Physical location of the epitopes. PAGDY epitope is depicted in blue, ITTANQY epitope is depicted in red. **B** Ribbon representation of the VP1 protein structure. **C** and **D** Structural model of the VP1 protein dimer (side and top views, respectively).

Finally, in order to analyze the degree of conservation of the newly identified epitopes, we aligned 181 different full length FCV capsid sequences (using Virus Pathogen Resource database). The non-neutralizing epitope was found to be highly conserved (Figure 4A). The sequence PAGDY (aa 431-435) was strictly conserved in 136 out of 181 sequences analyzed (75%). Residues P431, G433 and Y435 were 100% conserved, while A432 and D434 were 89% and 83% conserved, respectively. In contrast, the sequence corresponding to the neutralizing epitope ITTANQY (aa 445-451) was unique to the FCV Urbana strain used in this study (Figure 4A). Residues I445, T447 and Y451 were highly conserved (99%, 94% and 87%, respectively), while residues T446 and A448 were less conserved (58% and 50%, respectively) and residues N449 and Q450 were very variable (7% and 22%, respectively).

Both epitopes were localized within surface exposed loops of P2 subdomain (Figure 4B). The neutralizing epitope (ITTANQY) lies at the margins of the P2 domain dimer, while the non-neutralizing epitope (PAGDY) locates close to the P2 domain dimer interface (Figures 4C and D).

## Discussion

Infection with FCV is common in domestic cats and can result in acute upper respiratory tract disease. Highly virulent strains of FCV can lead to systemic disease with high mortality rates. Vaccination is therefore strongly recommended for all cats [24].

MAbs are efficient tools for detection and characterization of viruses and have wide applications as diagnostics, development of control measures, as well as for research in different aspects of their biology. Epitopes recognized by antibodies on viral capsids may overlap with other functional sites. Neutralizing antibodies bind specifically to exposed structures on the surface of the viral capsid and interfere with viral functions such as attachment, entry, or subsequent steps critical to infectivity [25]. Thus, precise identification of epitopes recognized by antibodies on virus capsids [26], along with structural determination of virus capsid-neutralizing antibody complexes [27, 28], can provide useful information, revealing specific mechanisms of neutralization and offering prospects for the development of efficient vaccines and therapeutics.

Here we have reported the generation and characterization of a panel of 26 MAbs against the VP1 capsid protein of FCV. Four MAbs recognized conformational epitopes, of which three were non-neutralizing and one was neutralizing. MAbs recognizing linear epitopes were subjected to epitope mapping analysis. Three non-neutralizing MAbs were found to bind epitopes within region F (amino acids 521-668). This was in agreement with findings of other groups, which had mapped the binding of linear non-neutralizing MAbs to this region [14, 17]. The epitopes recognized by the remaining 19 MAbs were all mapped to E5′HVR (aa 426 to 460), confirming previous studies indicating this 35 amino acid surface exposed region is a major antigenic determinant in FCV capsid [4, 5, 14, 15, 18]. Two close linear epitopes were identified: PAGDY (aa 431-435), recognized by 8 non-neutralizing MAbs and ITTANQY (aa 445-451), recognized by 11 neutralizing MAbs. Although previously reported linear neutralizing MAbs had not been precisely mapped, sequence analysis of MAb escape mutants identified point mutations located at amino acid positions: 441, 448, 449 and 455 [15], two of them forming part of epitope ITTANQY (Figure 3C), involving highly variable positions (Figure 4A), and the other two located in close proximity. Interestingly, the neutralizing epitope (aa 445-451) precisely matched the antigenic site 2 identified by Radford et al. [4], in a study focused on mapping linear B cell epitopes in FCV capsid protein recognized by feline polyclonal antisera derived from vaccinated or FCV-infected cats. The study showed the relevance of this epitope in the context of the immune response elicited by cats, the natural host for FCV, although it did not provide information regarding its neutralizing ability or significance in protection against virus infection. We now have determined the neutralizing activity of the MAbs binding to it. In fact, we have previously shown that chimeric RHDV VLPs displaying a 20-mer peptide derived from the FCV capsid protein (aa 440-459) encompassing the ITTANQY epitope, elicited a strong neutralizing immune response in groups of inoculated mice, which was similar to that elicited by native FCV VLPs [22]. The high sequence diversity observed at this epitope among the 181 FCV sequences analyzed, suggests that this region is under high immune selective pressure. Indeed, previous studies focused on evolutionary mechanisms of persistence and diversification of feline calicivirus found patterns of evolution associated with positive selection, suggesting an immune-mediated mechanism for viral evolution [29]. Codons predicted to be under positive selection mapped to E5′HVR and E3′HVR, including variable amino acid positions within ITTANQY epitope.

The neutralization mechanisms of the MAbs reported in this study have not been determined. However, the information available from previous reports suggests that binding of MAbs to the ITTANQY epitope (aa 445-451), might interfere with several events that take place upon virus attachment, involving this FCV capsid region. The binding of the VP1 dimer to fJAM-A receptor induces conformational changes. The VP1 surface loop at residues 435–447 (encompassing part of the epitope) undergoes structural rearrangement, rising towards the receptor [12]. Several hydrogen bonds are predicted to be formed between VP1 and fJAM-A, involving residues N443 and D444 (adjacent to the epitope) [12]. Subsequently, the minor structural protein VP2 forms a portal-like assembly that mediates viral endosome escape during first stages of viral infection [12]. Interactions are established between FCV proteins VP1 and VP2 involving VP1 residues: Y451, D452, A454 and D455 (forming part or in the vicinity of the epitope) [12]. Interestingly, a recent study focused on identifying specific molecular markers of VS-FCV strains, found seven residue positions to be statistically significant for pathotype differentiation [30], five of them located in E5′HVR region, residues: 438, 440, 448, 452 and 455, within or adjacent to the neutralizing epitope. Thus, the neutralizing MAbs reported in this study (those recognizing ITTANQY epitope, as well as 51C9 conformational MAb), represent suitable tools to be used in studies involving structural determination of FCV capsid-neutralizing antibody complexes, that might provide new insights regarding the characterization of the initial steps of FCV infection.

On the other hand, the non-neutralizing epitope PAGDY (aa 431-435), located close to the P2 dimer interface (Figures 4C and D), was found to be highly conserved among the full length FCV capsid sequences available (Figure 4A), despite forming part of a hypervariable region, E5HVR. Residues P431, G433 and Y435 forming part of this epitope were 100% conserved, suggesting mutations at these conserved sites of the capsid can compromise critical steps in the virus infectious life cycle. In fact, in a recent study, recombinant FCV viruses with mutations: P431A or G433A were shown to be nonviable. Furthermore, the corresponding mutated VP1 proteins did not form detectable VLPs [13], suggesting these surface residues are critical for FCV capsid assembly or stability. It is therefore expected that MAbs recognizing the surface exposed and highly conserved epitope PAGDY (aa 431-435) will react with most circulating FCV strains. Thus, these MAbs are broadly cross-reactive reagents that might be useful for diagnostic assays for FCV detection.

In summary, we have produced and extensively characterized a panel of MAbs specific for FCV capsid protein. These antibodies might be valuable for diagnostic applications, as well as for further research in different aspects of the biology of FCV.
